# Second Attempt of Cabergoline Withdrawal in Patients with Prolactinomas after a Failed First Attempt: Is it Worthwhile?

**DOI:** 10.3389/fendo.2015.00011

**Published:** 2015-02-04

**Authors:** Lucio Vilar, José Luciano Albuquerque, Patrícia Sampaio Gadelha, Frederico Rangel Filho, Aline Maria C. Siqueira, Maíra Melo da Fonseca, Karoline Frazão Viana, Barbara Sales Gomes, Ruy Lyra

**Affiliations:** ^1^Division of Endocrinology, Hospital das Clínicas, Federal University of Pernambuco, Recife, Brazil

**Keywords:** cabergoline, dopamine agonists, prolactinoma, second withdrawal, recurrence

## Abstract

Successful discontinuation of cabergoline (CAB) treatment has been reported in 31–75% of prolactinomas patients treated for at least 2 years. In contrast, it is not well established whether CAB therapy can be successfully withdrawn after a failed first attempt. This prospective open trial was designed to address this topic and to try to identify possible predictor factors. Among 180 patients with prolactinomas on CAB therapy, the authors selected those who fulfilled very strict criteria, particularly additional CAB therapy for at least 2 years, normalization of serum prolactin (PRL) levels following CAB restart, no tumor remnant >10 mm, no previous pituitary radiotherapy or surgery; and current CAB dose ≤1.0 mg/week. Recurrence was defined as an increase of PRL levels above the upper limit of normal. A total of 34 patients (70.6% female) treated with CAB for 24–30 months were recruited. Ten patients (29.4%) remained without evidence of recurrence after 24–26 months of follow-up. Twenty-four patients (70.6%) recurred within 15 months (75% within 12 months) after drug withdrawal and ~80% were restarted CAB. Median time to recurrence was 10.5 months (range, 3–15). Despite overlapping values, non-recurring patients had significantly lower mean PRL levels before withdrawal. Moreover, the recurrence rate was lower in subjects without visible tumor on pituitary magnetic resonance imaging (MRI) than in those with small remnant tumor (60 vs. 79%), though the difference was not statistically significant (*P* = 0.20). No other characteristic could be identified as a predictor of successful CAB discontinuation. In conclusion, a second attempt of CAB withdrawal after two additional years of therapy may be successful, particularly in patients with lower PRL levels and no visible tumor on pituitary MRI. Close monitoring of PRL level is mandatory, especially within the first year after withdrawal, where most recurrences are detected.

## Introduction

Prolactinomas are the most common pituitary tumors and account for about half of cases (1, 2). Their prevalence in the general population ranges from 6 to 50 per 100,000 (3, 4). Without appropriate treatment, prolactinomas may cause hypogonadism, infertility, bone loss, headaches, and visual fields defects from mass effect (5), as well as metabolic disorders (6). Based on their size, prolactinomas are classified into macro adenoma (>1 cm) or micro adenoma (<1 cm) (5). The mainstay of therapy involves the use of dopamine agonists (DA), even for patients with optic chiasm compression by large tumors (7, 8). Cabergoline (CAB) has been largely recommended as the first line agent, due to its better tolerability and higher efficacy in normalizing prolactin (PRL) levels and inducing tumor shrinkage, compared to bromocriptine (2, 5, 7, 8).

Studies of patients treated with CAB for microprolactinomas and macroprolactinomas have reported normalization of PRL levels in 75–90%, associated with an average decrease in tumor volume of 72–92% (9–11). In the Brazilian multicenter study on hyperprolactinemia, CAB therapy was able to induce significant tumor shrinkage and complete disappearance of tumor in 80 and 57.5% of patients, respectively (7).

A major drawback of DA therapy is the potential need to keep the medication indefinitely in many patients (2, 5). Indeed, despite the widespread use of DA for patients with prolactinomas and symptomatic idiopathic hyperprolactinemia for many decades, the optimal treatment strategy and duration of treatment is still not evident (12).

The 2011 Endocrine Society Clinical Practice Guideline recommends that, with careful clinical and biochemical follow-up, therapy may be tapered and perhaps discontinued in patients who have been treated with DA for at least 2 years, provided they no longer have elevated PRL levels, nor detectable tumor remnant on magnetic resonance imaging (MRI) (13). Accordingly, four recent studies (*n* = 292) have demonstrated that CAB therapy can be successfully discontinued, although hyperprolactinemia recurrence rates ranged from 25 to 69% (mean, 49%) (14–17).

On the other hand, it is not well established whether CAB therapy can be successfully withdrawn after a failed first attempt (18). This prospective study was conducted to address this topic and to try to identify possible predictor factors.

## Subjects and Methods

### Patients and Study Protocol

Among 180 patients with prolactinomas on CAB therapy routinely followed in the Division of Endocrinology, Hospital das Clínicas, Federal University of Pernambuco, Recife, Brazil, the authors selected those who fulfilled all of the following criteria: (1) recurrence of hyperprolactinemia after a first CAB withdrawal; (2) additional CAB therapy for at least 2 years; (3) normalization of serum PRL levels following CAB restart; (4) no tumor remnant ≥10 mm in its largest diameter on a recent (<6 months) MRI; (5) no previous pituitary radiotherapy or surgery; (6) no history of pregnancy over the past 3 years; (7) negative screening for macroprolactinemia; and (8) current CAB dose ≤1.0 mg/week.

After CAB withdrawal, the patients underwent clinical and hormonal (PRL levels) evaluation at 30 days and then every 3 months thereafter. Those patients who experienced disease recurrence, defined as an increase of PRL levels above the upper limit of normal (ULN) for gender, were removed from the study and restarted CAB at the same previous dose, according to the severity of their symptoms. They were also submitted to a pituitary MRI, aiming to detect any change in tumor remnant volume.

The study protocol was approved by our Local Ethics and Scientific Committees and all patients gave written informed consent.

### Hormone Assays and Imaging Studies

Serum PRL was measured using a two-site chemiluminescent immunometric assay. The interassay coefficient of variation was <5%. Normal range was 1.2–29.9 ng/ml (25–634 μUI/ml) for women and 2.6–18.1 ng/ml (55–384 μUI/ml) for men.

The radiological study included the evaluation of the sellar region by MRI with axial, coronal, and sagittal slices in T1, pre- and post-gadolinium, and in T2. The MRI was evaluated by a skilled neuroradiologist.

### Statistical Analysis

For comparison of categorical variables, the chi-squared test or the Fisher exact test were used where appropriate. A paired Student’s *t*-test was performed for the comparative analysis of quantitative variables. Results are expressed as percentages or mean values ± SD, unless otherwise stated. A stratified analysis was performed aiming at finding potential associations among clinical, imaging, and biochemical characteristics with outcome. Values of *P* < 0.05 were considered statistically significant. STATA version 10.0 and SPSS version 16.0 were used as statistical software.

## Results

### Characteristics of Patients

A total of 34 patients, 24 women and 10 men, fulfilled the selection criteria. Their individual baseline characteristics are shown in Table 1. At diagnosis, their mean age was 32.7 ± 4.53 years (range, 24(42; median, 32) and 11 (32.3%) had microadenomas. At withdrawal, their mean age was 41.35 ± 4.84 years (range, 31(52; median, 42) and they have been treated with CAB for 27.03 ± 2.02 months (range, 24(30; median, 27). The average weekly CAB dose at second withdrawal was 0.89 ± 0.20 mg (median, 1.0; range, 0.5(1.0) (Table 2).

**Table 1:** **Patients characteristics prior to the introduction of cabergoline therapy**.

Patient	Gender	Age (years)	PRL (ng/ml)^a^	Tumor largest diameter (cm)
1^b^	F	24	180	1.5
2^b^	F	34	660	2.8
3^b^	M	38	880	3.2
4^b^	F	26	312	0.8
5^b^	M	38	280	2.2
6^b^	M	30	94.7	0.9
7^b^	F	33	146	0.8
8^b^	F	30	172	0.8
9^b^	F	36	460	2.2
10^b^	F	26	214	1.8
11	F	28	420	2.2
12	M	38	140.5	0.9
13	F	36	255	1.2
14	M	36	124	1.2
15	M	32	334	1.5
16	F	31	720	2.1
17	F	38	95.2	0.8
18	M	35	177	0.8
19	M	37	335	1.6
20	M	37	240	1.2
21	F	34	212	0.9
22	M	36	910	3.5
23	F	34	163	0.9
24	F	38	382.4	1.6
25	F	36	412.5	1.8
26	F	35	265	0.9
27	F	37	511	2.1
28	F	24	223.4	1.3
29	F	30	242.7	1.2
30	F	27	712	2.5
31	F	35	180	0.8
32	F	30	310	1.7
33	F	33	256.3	1.3
34	F	40	314.4	1.4

*M, male; F, female; PRL, prolactin*.

*^a^Multiply by 21.2 to convert to micro international units per milliliter*.

*^b^Non-recurring patients after cabergoline withdrawal*.

**Table 2:** **Patients characteristics at cabergoline (CAB) therapy withdrawal**.

Patient	Age (years)	PRL (ng/ml)^a^	Tumor remnant largest diameter (cm)	CAB dose (mg/week)	Duration of CAB therapy (months)	Time for recurrence after first withdrawal (months)
1^b^	32	9.6	NVT	0.5	24	9
2^b^	44	18.3	NVT	1	25	15
3^b^	52	15.8	0.5	1	30	6
4^b^	38	13.5	NVT	1	28	8
5^b^	42	15.3	NVT	1	28	9
6^b^	38	8.2	0.4	0.5	27	6
7^b^	40	12.5	NVT	0.5	29	12
8^b^	43	20.8	NVT	1	26	15
9^b^	39	22.5	0.5	1	30	9
10^b^	33	20.8	0.4	1	24	12
11	36	23.2	0.6	1	25	6
12	47	9.6	0.4	0.5	30	9
13	43	22.5	NVT	1	28	6
14	43	14.8	NVT	1	28	6
15	40	16.8	0.6	1	30	6
16	39	22.4	NVT	1	29	6
17	48	15.7	NVT	0.5	26	9
18	43	16	NVT	0.5	27	6
19	45	17.5	0.5	1	30	6
20	45	17.1	NVT	1	24	15
21	42	23.4	0.4	1	25	9
22	46	17.7	0.9	1	28	6
23	42	22.7	NVT	0.5	28	9
24	47	20.4	0.6	1	26	3
25	42	22.3	0.5	1	27	15
26	43	21.4	0.4	1	29	9
27	45	18.2	0.5	1	26	9
28	31	19.2	NVT	1	30	6
29	37	22.3	0.4	1	26	6
30	34	24.6	0.5	1	26	6
31	42	18.2	NVT	1	26	9
32	37	25.2	0.5	1	25	6
33	40	22.2	0.4	1	24	15
34	48	21.8	0.5	1	25	9

*NVT, no visible tumor; PRL, prolactin*.

*^a^Multiply by 21.2 to convert to micro international units per milliliter*.

*^b^Non-recurring patients after cabergoline withdrawal*.

Among the 146 patients not enrolled in the study, many presented with at least two exclusion criteria. The most prevalent were weekly CAB dosage >1 mg (60%), treatment duration <2 years (43%), tumor remnant >1 cm (16%), and concomitant macroprolactinemia (10%).

### Responsiveness to Cabergoline Withdrawal

**Figure 1:**
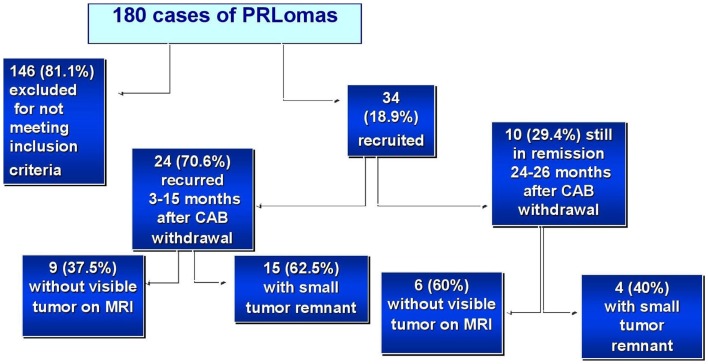
**Characteristics and behavior of the patients with prolactinomas (PRLomas) who were evaluated**.

**Figure 2:**
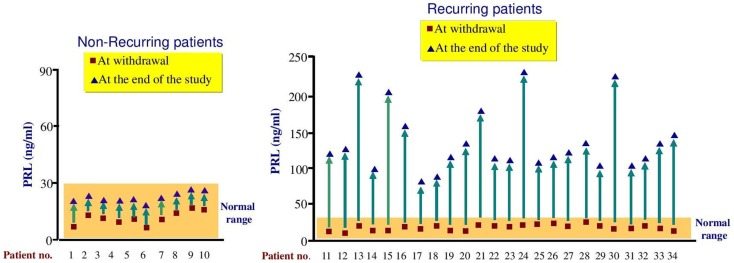
**Comparison of PRL levels in non-recurring and recurring patients at withdrawal and at the end of the study**. Recurrence was defined as PRL values above the upper limit of normal.

The median follow-up time was 12 months (ranging from 3 to 26 months). Overall, 24 out of 34 patients (70.6%) recurred within 15 months after CAB discontinuation (Figures 1 and 2). Eighteen patients (75%) did so within 12 months, 11 (45.8%) within 9 months, 7 (29.2%) within 6 months, and 2 (8.3%) within 3 months, respectively. Six patients (25%) relapsed after 12 months of follow-up (Figures 1 and 2). Median time to recurrence was 10.5 months. The follow-up for patients who recurred was considered complete at the time of recurrence. Noteworthy, none of the patients reached PRL levels above those found before CAB therapy introduction. Thus, mean PRL levels were significantly lower at the end of the follow-up (132.52 ± 48.3 vs. 366.88 ± 234.14 ng/ml, *P* < 0.01).

Among recurring female patients, four presented with amenorrhea, eight with amenorrhea and galactorrhea, whereas five remained asymptomatic. Concerning recurring male patients, all developed hypogonadism-related symptoms. None of the tumors enlarged in the patients experiencing recurrence. CAB was restarted in all symptomatic recurring patients.

A total of 10 patients (29.4%) remained without clinical and biochemical evidence of hyperprolactinemia recurrence after a median follow-up time of 24 months (range, 24–26 months). However, as shown in Figure 2, some increase in PRL levels was observed in all these patients, though they remained in the normal range.

### Predictors of Responsiveness to Cabergoline Withdrawal

When recurring and non-recurring patients were compared, no significant difference was found concerning age, gender, CAB dose, and duration of CAB therapy before the second withdrawal (Table 3). Moreover, the proportion of patients with micro- (29.2 vs. 40%) and macroadenomas (70.8 vs. 60%) was similar in both groups (*P* = 0.40) (Table 3), and so was their initial tumor size (1.47 ± 0.65 vs. 1.7 ± 0.89 cm, *P* = 0.45) (Table 4). Likewise, the mean time of recurrence after first CAB withdrawal did not significantly differ (10.1 ± 3.28 vs. 8.0 ± 3.14 months; *P* = 0.66).

**Table 3:** **Clinical, biochemical, and imaging characteristics in recurring and non-recurring patients at withdrawal**.

	Recurring patients (*n* = 24)	Non-recurring patients (*n* = 10)	*P*-value
Age (years)
Range	31–48	32–52	0.64^a^
Mean ± SD	41.87 ± 4.55	40.10 ± 5.72	
Median	42.5	39.5	
Female gender	17/24 (70.8%)	7/10 (70.0%)	0.63^b^
Male gender	7/24 (29.2%)	3/10 (30.0%)	0.63^b^
PRL (ng/ml)^c^
Range	9.6–25.2	8.2–22.5	0.01^a^
Mean ± SD	19.8 ± 3.70	15.73 ± 4.87	
Median	20.9	15.55	
CAB dose before withdrawal (mg/week)
Range	0.5–1.0	0.5–1.0	0.41^a^
Mean ± SD	0.91 ± 0.19	0.85 ± 0.24	
Median	1	1	
CAB therapy duration before withdrawal (months)
Range	24–30	24–30	0.85^a^
Mean ± SD	27 ± 1.96	27.1 ± 2.28	
Median	26.5	27.5	
Pituitary mri findings
No visible tumor	9 (37.5%)	6 (60.0%)	0.20^b^
Tumor remnant <1 cm	15 (62.5%)	4 (40.0%)	

*CAB, cabergoline; PRL, prolactin; MRI, magnetic resonance imaging*.

*^a^Student’s t-test*.

*^b^Fisher’s exact test*.

*^c^Multiply by 21.2 to convert to micro international units per milliliter*.

**Table 4:** **Clinical, biochemical, and imaging characteristics in recurring and non-recurring patients at diagnosis**.

	Recurring patients (*n* = 24)	Non-recurring patients (*n* = 10)	*P*-value
Age (years)
Range	24–40	24–38	0.34^a^
Mean ± SD	34.04 ± 3.96	31.50 ± 5.10	
Median	35	31.5	
Female gender	17/24 (70.8%)	7/10 (70.0%)	0.63^b^
Male gender	7/24 (29.2%)	3/10 (30.0%)	0.63^b^
PRL (ng/ml)^c^
Range	95.2–910	94.7–880	0.10^a^
Mean ± SD	330.64 ± 203.13	339.87 ± 253.92	
Median	260.65	247	
MRI findings
Microadenomas	7 (29.2%)	4 (40%)	0.4^b^
Macroadenomas	17 (70.8%)	6 (60%)	

*CAB, cabergoline; PRL, prolactin; MRI, magnetic resonance imaging*.

*^a^Student’s t-test*.

*^b^Fisher’s exact test*.

*^c^Multiply by 21.2 to convert to micro international units per milliliter*.

By contrast, non-recurring patients had significantly lower mean PRL levels (15.73 ± 4.87 vs. 19.8 ± 3.70 ng/ml, respectively, *P* < 0.01) before second withdrawal (Table 3). Nevertheless, there was a high degree of overlap in PRL values in both groups (Table 3). Moreover, the recurrence rate was lower in subjects without visible tumor on pituitary MRI than in those with small remnant tumor (60 vs. 79%), though the difference was not statistically significant (*P* = 0.20).

Noteworthy, demographic, biochemical, and imaging features at the prolactinoma diagnosis did not significantly differ when patients who recurred and the ones who remained in remission were compared (Table 4).

## Discussion

The optimal duration of therapy with DA for patients with prolactinomas or non-tumoral hyperprolactinemia remains controversial (12). There is however strong and growing evidence that discontinuation of dopamine agonist treatment may be successfully achieved in a selected group of patients treated for at least 2 years (14–17, 19, 20). A recent systematic review and meta-analysis has shown that the pooled proportion of patients with persisting normoprolactinemia after CAB withdrawal was 35% in a random effects model (21). In the series by Karlip et al. (15), which involved 46 patients, the overall recurrence was 54% and the median time to recurrence was 3 months (range, 1(18 months) (15).

In contrast, it is currently unknown how valid and how useful could be a second try to discontinue CAB administration in patients whose first withdrawal did not succeed. Presently, there are only data from a recent pilot prospective two-center study that evaluated 17 patients who had undertaken a second course of CAB treatment for at least 24 additional months (18). During a median follow-up of 6.1 months (ranging from 1 to 60 months) after CAB withdrawal, 11 patients (64.7%) recurred. The estimated overall recurrence rate was 44 events per 100 person-years. Moreover, the estimated cumulative hazard of recurrence was 40% at 6 months and 82% at 12 months (18).

We conducted a prospective trial to evaluate the outcome of a second attempt of CAB withdrawal in 34 patients who met strict selection criteria and have been treated with CAB for 24(30 months (median, 27 months). All patients presented with normal PRL levels, were given CAB doses (1 mg weekly, and had either no visible tumors or small tumor remnants (<1 cm) on MRI. We found that 10 patients (29.4%) were able to maintain normal PRL levels for up to 26 months following CAB withdrawal.

Concerning the 24 recurring subjects, all recurred within 15 months after CAB withdrawal, whereas 18 (75%) did so within 12 months. The median time to recurrence was 10.5 months. Similarly, in the study by Kwancharoen et al. (18), most of the recurrence (59%) also occurred within 1 year, whereas the median time to recurrence was 6 months. Likewise, Karlip et al. (15) had previously reported that 91% of the recurrences after the first withdrawal were observed within the first year of discontinuation. Thus, close clinical monitoring, especially within the first year after withdrawal, should be carried out in all patients in whom CAB therapy is discontinued.

According to previous studies, predictor factors for higher chance of successful CAB withdrawn include lower PRL levels, longer duration of treatment, tumor size (micro- > macroadenomas), previous pituitary radiotherapy or surgery, and pregnancy (5, 12, 13, 15, 16, 22). It has been shown that women with prolactinomas who became pregnant have a higher rate of remission than women without previous pregnancy (22, 23). Moreover, PRL levels are lower after delivery as compared to levels before conception and complete remission of hyperprolactinemia has been reported in 17–37% of women after pregnancy (23). For that reason, we did not include in our study women with history of pregnancy over the last 3 years.

Among our patients, a stratified analysis on clinical, pituitary imaging, and biochemical characteristics, at the beginning of the study and at CAB withdrawal, was unable to detect parameters that could quite accurately identify individuals most likely to respond to treatment discontinuation. However, non-recurring patients had significantly lower mean PRL levels at withdrawal. Furthermore, the recurrence rate was lower in patients without visible tumor remnant on pituitary MRI than in subjects with small tumor remnants (60 vs. 79%), though the difference was not statistically significant (*P* = 0.20). Accordingly, Hu et al. (21) reported that patients who received the lowest CAB dose and presented a significant reduction in tumor size before withdrawal were more likely to achieve the best success (*P* < 0.001). Likewise, in the series by Karlip et al. (15), size of tumor remnant prior to withdrawal predicted recurrence (18% increase in risk for each millimeter). By contrast, Kwancharoen et al. (18) could not depict any statistically significant clinical predictors of recurrence, but this may have been influenced by the small number of subjects enrolled in their trial (*n* = 17).

It was previously reported that a nadir PRL at the time of first withdrawal below 5.4 ng/ml was associated with a lower risk of recurrence (16). In our series, although mean PRL levels before withdrawal were lower in non-recurring subjects, individual PRL values in both groups greatly overlapped (8.2–22.5 vs. 9.6–25.2), as shown in Table 3.

Our study has some limitations. Notably, because of the small number of patients, some or most clinical predictors may not have reached statistically significance. It is also possible that some of the patients presently on remission may recur later on. However, recurrences beyond 2 years after CAB withdrawal have only seldom been reported (15–17, 24). In the largest series to date (*n* = 200), 63 patients (31.5%) had recurrent hyperprolactinemia, 56% during the first year, 33% during the second year, 11% during the third year, and none thereafter (*P* < 0.001) (16, 24).

As shown in Table 5, a combined analysis of our findings and those from the study by Kwancharoen et al. (18) would indicate that about 31% of a selected group of patients with prolactinomas treated with CAB for at least additional 24 months could benefit from a second drug discontinuation. In four recent previous studies, which involved 292 patients with micro- or macroprolactinomas, the mean recurrence rate after first CAB withdrawal was 49% [range, 25–69% (14–16)]. Therefore, the chance of achieving a successful second withdrawal seems to be similar to the first one. However, further studies with a greater number of patients and longer-term follow-up are clearly warranted.

**Table 5:** **Data from two studies specifically designed to evaluate the likelihood of successful second cabergoline (CAB) withdrawal after a failed first attempt in well-controlled patients with prolactinomas**.

Authors	Number of patients evaluated	Definition of recurrence	Number of non-recurring patients after withdrawal	Number of recurring patients after withdrawal	Median time to recurrence	Recurrence rate within 1 year after CAB discontinuation (%)	Duration of treatment	Duration of follow-up after withdrawal
Kwancharoen et al. (19)	17	PRL level > ULN for gender and age	6 (35.3%)	11 (64.7%)	6 months	59	24–93 months (median, 48)	1–60 months (median, 6.1)
Current study	34	PRL level > ULN for gender	10 (29.4%)	24 (70.6%)	10.5 months	75	24–30 months (median, 27)	3–26 months (median, 12)
All	51	–	16 (31.4%)	35 (68.6%)	–	68.6	–	–

A likely explanation for the successful second withdrawal could be a longer exposure to CAB therapy and consequently to its antiproliferative and pro-apoptotic effects on pituitary tumor cells, described in several studies (25).

An additional advantage of CAB withdrawal would be to reduce the risk of fibrotic cardiac valvulopathy, which has been associated with the use of DA therapy (26–28), particularly with the higher dosage given to Parkinson’s disease patients (29, 30). However, most studies have not shown an increased prevalence of significant cardiac valve regurgitation in patients with pituitary diseases receiving CAB (13, 31–33).

In conclusion, the findings of the current study yield increasing awareness that a second trial of CAB discontinuation should be attempted in well-selected patients with prolactinomas who display normal PRL levels and tumor remnant <1 cm at low doses (≤1 mg/week). Although our patients have been treated for two or more years, the optimal length of therapy before attempting a second CAB withdrawal still needs to be established. Patients with lower PRL levels and no visible tumor on MRI seemed to be the most likely to benefit from this approach but it was also effective in subjects with small tumor remnants. It is essential however to closely follow-up these patients and reinstitute therapy promptly in symptomatic recurrent ones.

## Conflict of Interest Statement

The authors declare that the research was conducted in the absence of any commercial or financial relationships that could be construed as a potential conflict of interest.
